# Detection of Vascular Reactive Oxygen Species in Experimental Atherosclerosis by High-Resolution Near-Infrared Fluorescence Imaging Using VCAM-1-Targeted Liposomes Entrapping a Fluorogenic Redox-Sensitive Probe

**DOI:** 10.1155/2021/6685612

**Published:** 2021-03-09

**Authors:** Simona-Adriana Manea, Mihaela-Loredana Vlad, Daniela Rebleanu, Alexandra-Gela Lazar, Ioana Madalina Fenyo, Manuela Calin, Maya Simionescu, Adrian Manea

**Affiliations:** Institute of Cellular Biology and Pathology “Nicolae Simionescu” of the Romanian Academy, Bucharest, Romania

## Abstract

Excessive production of reactive oxygen species (ROS) and the ensuing oxidative stress are instrumental in all phases of atherosclerosis. Despite the major achievements in understanding the regulatory pathways and molecular sources of ROS in the vasculature, the specific detection and quantification of ROS in experimental models of disease remain a challenge. We aimed to develop a reliable and straightforward imaging procedure to interrogate the ROS overproduction in the vasculature and in various organs/tissues in atherosclerosis. To this purpose, the cell-impermeant ROS Brite™ 700 (RB700) probe that produces bright near-infrared fluorescence upon ROS oxidation was encapsulated into VCAM-1-targeted, sterically stabilized liposomes (VLp). Cultured human endothelial cells (EC) and macrophages (Mac) were used for *in vitro* experiments. C57BL6/J and ApoE-/- mice were randomized to receive normal or high-fat, cholesterol-rich diet for 10 or 32 weeks. The mice received a retroorbital injection with fluorescent tagged VLp incorporating RB700 (VLp-RB700). After two hours, the specific signals of the oxidized RB700 and 1,2-distearoyl-sn-glycero-3-phosphoethanolamine-N-(7-nitro-2-1,3-benzoxadiazol-4-yl) (NBD-DSPE), inserted into liposome bilayers, were measured *ex vivo* in the mouse aorta and various organs by high-resolution fluorescent imaging. VLp-RB700 was efficiently taken up by cultured human EC and Mac, as confirmed by fluorescence microscopy and spectrofluorimetry. After systemic administration in atherosclerotic ApoE-/- mice, VLp-RB700 were efficiently concentrated at the sites of aortic lesions, as indicated by the augmented NBD fluorescence. Significant increases in oxidized RB700 signal were detected in the aorta and in the liver and kidney of atherosclerotic ApoE-/- mice. RB700 encapsulation into sterically stabilized VCAM-1-sensitive Lp could be a novel strategy for the qualitative and quantitative detection of ROS in the vasculature and various organs and tissues in animal models of disease. The accurate and precise detection of ROS in experimental models of disease could ease the translation of the results to human pathologies.

## 1. Introduction

Reactive oxygen species (ROS) play a major role in cell physiology. However, when produced in excess in response to various pathological stimuli, ROS are harmful molecules causing cell damage by complex mechanisms that generally involve activation of specific signal transduction pathways and reversible/irreversible oxidation-induced alterations of biological molecules (e.g., lipids, proteins, nucleic acids, and carbohydrates) [1].

ROS are chemically reactive oxygen-derived molecules that result as by-products of cellular respiration and metabolism and are produced also in a highly regulated manner by specific enzymatic systems. One- or two-electron reduction of molecular oxygen (O_2_) gives rise to superoxide anion (O_2_^·-^) formation and hydrogen peroxide (H_2_O_2_), respectively. Both O_2_^·-^ and H_2_O_2_ are implicated in secondary chemical reactions to produce highly toxic free radicals such as hydroxyl radical (HO^·^), hydroperoxyl radical (HOO^·^), and peroxynitrite anion (ONOO^−^). Cells and organisms have evolved to limit the potentially deleterious effects of ROS by expressing highly organized enzymatic and nonenzymatic antioxidant systems. Yet, unlike O_2_^·-^ and H_2_O_2_ that are specifically scavenged by dedicated antioxidant enzymes (i.e., superoxide dismutase and catalase), HO^·^, HOO^·^, and ONOO^−^ cannot be neutralized by enzymatic reactions. Thus, the latter ROS readily interact with biological molecules at their site of formation and generally trigger a chain of oxidation reactions, further contributing to the aggravation of cellular oxidative stress. Collectively, being highly reactive and relatively short-lived molecules (10^−9^ to 10^−6^ seconds), the type, the rate of formation, and the intracellular compartmentalization of ROS are likely to dictate their physiological or pathological effects on biological systems [2].

ROS overproduction and the ensuing oxidative stress have been mechanistically linked to numerous human pathologies including cardiovascular diseases (CVD), metabolic disorders, cancer, and neurodegeneration. Others and we have demonstrated that augmented formation of ROS is instrumental in all phases of atherosclerosis [3], and it is a hallmark of CVD, in general [4]. A strong correlation between upregulated ROS and the severity of atherosclerotic lesions has been demonstrated in humans and atherosclerotic mice [3–6]. Notably, early atherosclerotic lesions can be barely detected using standard imaging procedures (e.g., echography and angiography) due to the fact that several parameters such as luminal diameter and vessel wall thickness are not essentially modified as compared to normal conditions. However, the early-stage lesions are characterized by robust oxidative and inflammatory reactions that further contribute to atheroma progression [7]. Thus, the need for novel tools to detect ROS formation in atherosclerosis cannot be overemphasized.

Recently, a novel water-soluble redox-sensitive fluorescent probe, ROS Brite™ 700 (RB700), has been developed and optimized for *in vivo* detection of ROS in small experimental animals by the AAT Bioquest Company (USA) (https://www.aatbio.com). The major advantage of RB700 is that it generates bright near-infrared fluorescence upon ROS oxidation; this important feature minimizes the artifacts typically caused by the interferences with the tissue autofluorescence and consequently results in a highly improved signal-to-background noise ratio. Yet, a shortcoming of this probe arises from its cell-impermeant attribute and therefore the intracellular and in particular the atherosclerotic plaque oxidative microenvironment is easily underestimated. To overcome this technical limitation, in an attempt to deliver and concentrate the probe at the atherosclerotic lesions, we designed a specific formulation of polyethylene glycol- (PEG-) stabilized liposomes (Lp) targeted towards vascular cell adhesion molecule 1 (VCAM-1) as nanocarriers for RB700. We provide here evidence that redox-sensitive imaging probe RB700 entrapped into VCAM-1-targeted PEGylated Lp localize reliably and quantify directly the formation of ROS within the atheromatous plaque and in various organs in experimental atherosclerosis.

## 2. Materials and Methods

### 2.1. Materials

Unless otherwise indicated, standard chemicals and reagents were obtained from Sigma-Aldrich. Lipids were purchased from Avanti Polar Lipids (Alabaster, AL, USA). VCAM-1 targeting peptide (NH_2_-VHPKQHRGGSKGC-COOH) was synthesized by GeneCust (Dudelange, Luxembourg). ROS Brite™ 700 (RB700) was obtained from AAT Bioquest. Primary and secondary antibodies were from Santa Cruz Biotechnology. American Type Culture Collection- (ATCC-) derived human umbilical vein endothelial cell (EC) line (EA.hy926) and human THP-1 monocytic (Mon) cell line were used.

### 2.2. Preparation and Characterization of VCAM-1-Targeted Liposomes as Nanocarriers of RB700 (VLp-RB700)

Sterically stabilized VCAM-1-targeted liposomes (VLp) were prepared using the extrusion method as we previously described [8]. Briefly, the basic components of the Lp were as follows: 45 mols% 1,2-dipalmitoyl-sn-glycero-3-phosphocholine (DPPC), 28 mols% cholesterol, 20 mols% 1,2-dioleoyl-snglycero-3-phosphatidic acid (DOPA), 2 mols% (1,2-dipalmitoyl-sn-glycero-3-phosphoethanolamine-N-(7-nitro-2-1,3-benzoxadiazol-4-yl) (ammonium salt) (NBD-PE), 4 mols% 1,2-distearoyl-sn-glycero-3-phosphoethanolamine-N-[methoxy (polyethylene glycol)-2000] (ammonium salt) (PEG-DSPE), and 1 mol% 1,2-distearoyl-sn-glycero-3-phosphoethanolamine-N-[maleimide (polyethylene glycol)-2000] (Mal-PEG-DSPE). The mixture of phospholipids in chloroform was dried in a rotary evaporator, and subsequently, the lipid film was hydrated with PBS or with a solution containing the fluorogenic redox-sensitive probe RB700. Then, the multilamellar vesicle suspension was extruded through 200 nm polycarbonate membranes using a Mini-Extruder (Avanti Polar Lipids, Alabaster, AL/USA). The peptide NH_2_-VHPKQHRGGSKGC-COOH, with a known affinity for VCAM-1 [9], was coupled to maleimide functionalized PEG lipids (Mal-PEG-DSPE) as described in [8]. Lp were centrifuged using Amicon™ Ultra 100 K filter units to separate the encapsulated from free RB700 and coupled peptide from uncoupled Lp. The fluorescence of RB700 in the filtrate of separation columns (free RB700) was measured at *λ*em = 706 nm (excitation *λ*ex = 680 nm) using a fluorospectrometer (NanoDrop™ 3300 Fluorospectrometer, Thermo Fisher Scientific) and used to determine the free RB700 concentration (from a standard curve of fluorescence for RB700 concentrations). The concentration of Lp-encapsulated RB700 was determined by subtracting free RB700 from the initially added concentration. The amount of VCAM-1 peptide coupled to the surface of Lp was determined employing ultra-high-performance liquid chromatography (UHPLC) analysis (Agilent 1290 Infinity, Agilent Technologies, Santa Clara, California, USA) as detailed in [8]. Particle size and polydispersity index (PDI) were analyzed by a dynamic light scattering (DLS) method on a Nano ZS apparatus (ZEN 3600) (Malvern Instruments, Malvern, UK).

### 2.3. Cell Culture

EC were grown to confluency in Dulbecco's modified Eagle's medium containing 10% fetal bovine serum (FBS) in accordance with the provider's instructions (ATTC). Mon-to-macrophage (Mac) differentiation was done by incubating THP-1 Mon for 3 days with 100 nmol/L phorbol 12-myristate 13-acetate (PMA) as we previously described [5].

### 2.4. *In Vitro* Cytotoxicity Analysis of VCAM-1-Targeted Lp Entrapping RB700

The potential toxic effects of VLp-RB700 were tested on cultured EC and Mac by the Vybrant™ MTT [3-(4,5-dimethylthiazole-2-yl)-2,5-diphenyltetrazolium bromide] cell proliferation assay in accordance with the manufacturer's protocol (Thermo Scientific).

### 2.5. Strategy for ROS Imaging in Experimental Atherosclerosis Model

Female B6.129P2-Apoetm1Unc/J (ApoE-/-; Stock No: 002052) and C57BL/6 (Stock No: 000664) mice were purchased from The Jackson Laboratory, bred in our facility under SPF conditions, and exposed to 12 h of light/dark cycles and with access to standard rodent diet and water *ad libitum*. To develop aortic atherosclerotic lesions [10], at 12 weeks of age, the ApoE-/- female mice were randomized (*n* = 15/group) to receive normal diet (ND) or high-fat, cholesterol-rich diet (HD) for 10 or 32 weeks. We had three experimental groups: (i) ApoE-/- mice that received ND for 10 weeks, (ii) ApoE-/- mice that received HD for 10 weeks, and (iii) ApoE-/- to which HD was administrated for 32 weeks. The C57BL6/J female mice fed a normal diet (*n* = 15) for 10 weeks were used as controls. The VLp-RB700 were delivered to mice via one retroorbital injection, under ketamine-xylazine-acepromazine (80/10/2 mg/kg) anesthesia, at a dose of 20 mM Lp/500 *μ*M RB700 (100 *μ*L bolus). After two hours, the animals were sacrificed and ≈1 mL of blood was collected via cardiac puncture. To remove any trace of circulating VLp-RB700, the animals were perfused (15 min) with warmed calcium-containing PBS, pH 7.4. The aortic perivascular adipose tissue was gently removed prior to the fluorescence analysis. The fluorescent signal was monitored *ex vivo* in the aorta and various organs, using the IVIS™ imaging system. To delineate the specific RB700 signal from the tissue autofluorescence, we performed spectral unmixing analysis using the Living Image 4.3.1 software. Oil Red O (ORO) staining was employed to assess aortic atherosclerotic lesions as we previously described [6]. The animal studies were conducted in agreement with the guidelines of EU Directive 2010/63/EU with the prior approval of the ethical committee of the Institute of Cellular Biology and Pathology “Nicolae Simionescu.”

### 2.6. Western Blot Assay

Aortic protein extracts and Western blot analysis were done as previously described [11]. Briefly, the entire aortas derived from C57BL6/J and ApoE-/- mice were suspended in RIPA buffer containing a protease inhibitor cocktail (Sigma) and subjected to glass bead-based (1.0 mm diameter) mechanical homogenization/disruption (BioSpec). Protein denaturation was done in Laemmli's electrophoresis sample buffer (Serva) at 95°C for 20 minutes. Protein samples (30 *μ*g/lane) were subjected to SDS-PAGE and transferred onto nitrocellulose membranes (Bio-Rad). The latter were incubated for 12 hours (4°C) with anti-nitrotyrosine (NT) (mouse monoclonal, sc-32757, dilution 1 : 200) primary antibody followed by one-hour exposure (room temperature) to an anti-mouse IgG-HRP (sc-2031, dilution 1 : 2000) as secondary antibody. Protein bands were detected by chemiluminescence imaging (ImageQuant LAS 4000 system, Fujifilm). TotalLab™-based densitometric analysis of the NT-protein adduct levels was done using the expression level of *β*-actin protein as internal normalization.

### 2.7. Statistical Analysis

Data derived from at least three independent experiments were expressed as the mean ± standard deviation. Statistical analysis was done by a *t*-test and one-way analysis of variance followed by Tukey's *post hoc* test; *P* < 0.05 was considered statistically significant.

## 3. Results

### 3.1. Characterization of VCAM-1-Targeted Lp (VLp)

The average hydrodynamic diameter of VLp was measured by DLS after 1 : 100 dilution in distilled water; the value obtained was 175 ± 30 nm (*n* = 9) with a unimodal size distribution, shown by the polydispersity index that was always smaller than 0.2. To target the activated endothelium, characteristically covering the atherosclerotic lesions, we took advantage of the surface-exposed VCAM-1 that is typically expressed by endothelial cells under inflammatory conditions [12]. As determined by UHPLC, the coupling efficiency of the specific VCAM-1 recognition peptide covalently linked to the liposomal bilayer was ≈10 *μ*g peptide/*μ*mol liposomes.

### 3.2. VCAM-1-Targeted Lp Are Efficiently Taken Up by Cultured Human EC and Mac

**Figure 1:**
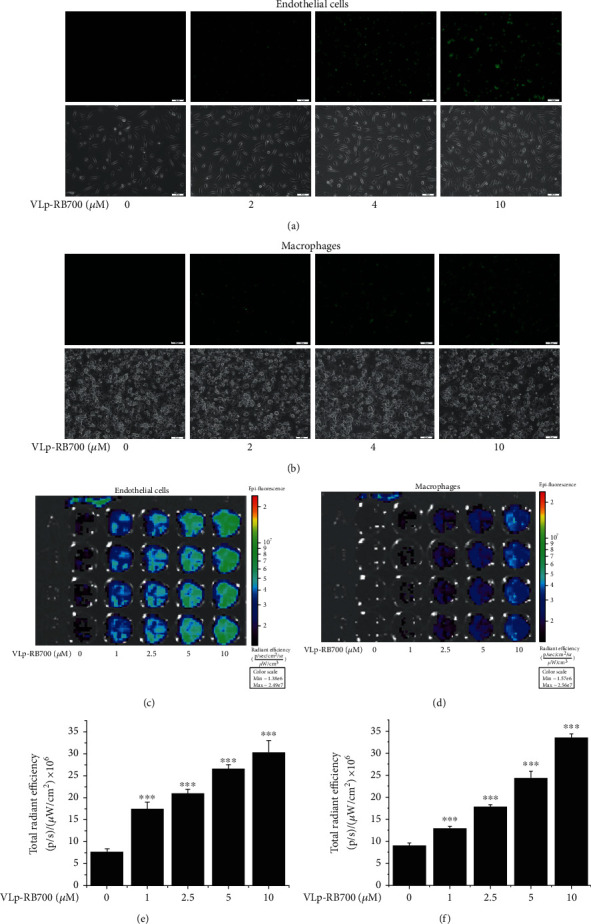
VCAM-1-targeted liposomes loaded with RB700 (VLp-RB700) are efficiently taken up by human endothelial cells (EC) and macrophages (Mac). Cultured cells were incubated for 2 hours with increasing concentrations of VLp-RB700 (1-10 *μ*M) and examined by phase contrast/fluorescence microscopy and high-resolution fluorescence imaging (IVIS™) techniques. (a, b) Qualitative analysis of the uptake of VLp-RB700 (NBD-DSPE, spectral range: green) was done by fluorescence microscopy (20x magnification, top panel). The corresponding phase contrast images are shown in the lower panel. Quantitative determination done with the IVIS™ system showing the concentration-dependent uptake of VLp-RB700 by (c, e) EC and (d, f) Mac. *n* = 4, ^∗∗∗^*P* < 0.001. Statistical *P* values were calculated in relation to the basal fluorescence emission level detected in the absence of Lp.

Endothelial cells (EC) and THP-1-derived Mac were employed to investigate the potential of VLp-RB700 nanocarrier to deliver RB700 into the cells. Cultured cells were exposed to increasing concentrations of VLp-RB700 (1-10 *μ*M) for 2 hours, then washed with fresh culture medium, and subjected to fluorescence-based assays. A concentration-dependent increase in the uptake of VLp-RB700 by cultured EC and Mac was detected by fluorescence microscopy (Figures 1(a) and 1(b)). Quantification of cellular internalization of VLp-RB700 that was done by high-resolution fluorescence analysis (IVIS™ Caliper, Perkin Elmer) demonstrated a steady, VLp-RB700 concentration-dependent significant increases in NBD-DSPE-labeled Lp fluorescence signal, both in cultured EC and Mac (Figures 1(c) and 1(e) and Figures 1(d) and 1(f)).

### 3.3. VLp-RB700 Are Not Cytotoxic to Human EC and Mac

**Figure 2:**
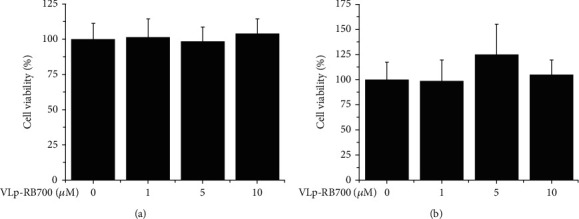
VLp-RB700 does not affect the viability of cultured (a) EC and (b) Mac. Human cultured EC and Mac were exposed for 24 hours to increasing concentrations of RB700-Lp (1-10 *μ*M). Cell viability was tested by the MTT method. *n* = 4.

As revealed by the MTT assay, VLp-RB700 was not cytotoxic and did not affect the viability of cultured EC and Mac after 24-hour incubation of the cells with increasing concentrations of VLp-RB700 (1-10 *μ*M) (Figures 2(a) and 2(b)).

### 3.4. Upregulation of Oxidized RB700 Near-Infrared Fluorescent Emission in the Presence of Fenton's Reagent

**Figure 3:**
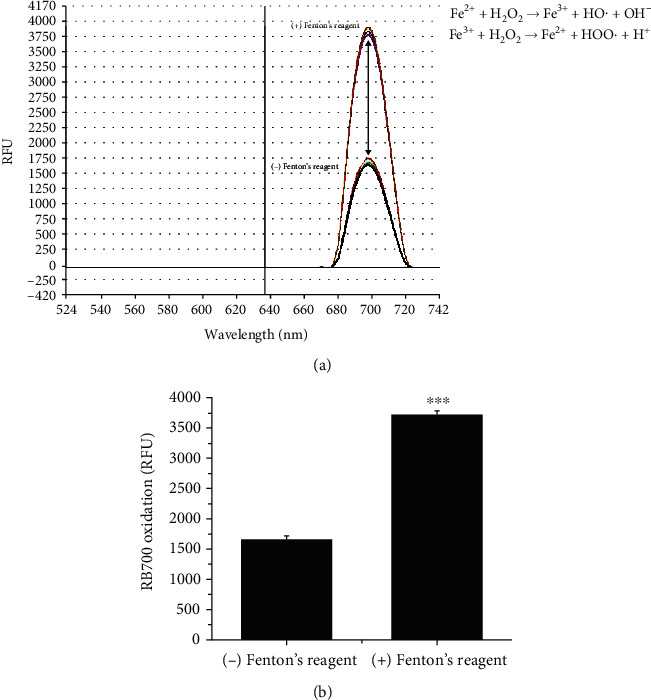
Test for the RB700 oxidation in the presence of Fenton's reagent. (a) The RB700 spectral analysis was done in the absence/presence of Fenton's reagent. Note the increase of the fluorescent emission intensity of RB700 due to the oxidation in the presence of ROS/HO^·^. (b) The absolute fluorescence emission (*λ*em = 706 nm) was quantified. *n* = 3 independent reactions. ^∗∗∗^*P* < 0.001. *P* values were calculated in relation to the basal fluorescence emission level detected in the absence of Fenton's reagent.

In order to establish the sensitivity and limit of detection of the method, we performed quantitative spectrofluorimetric analysis of RB700 oxidation in the presence of Fenton's reagent, as a source of HO^·^. Briefly, RB700 (25 *μ*M) was oxidized in the presence of Fenton's reagent (10 *μ*M FeSO_4_ + 100 *μ*M H_2_O_2_) for 1 minute and then subjected to spectral analysis of the ROS-modulated RB700 fluorescence emission. As depicted in Figure 3, the RB700 indicator showed a basal fluorescence signal in the absence of Fenton's reagent that led to a significant increase in peak shifting (≈2.3-fold) of the near-infrared fluorescence emission upon ROS/HO^·^ oxidation.

### 3.5. Establishing the Optimal Detection Conditions of Oxidized RB700 Using the IVIS™ Imaging System

**Figure 4:**
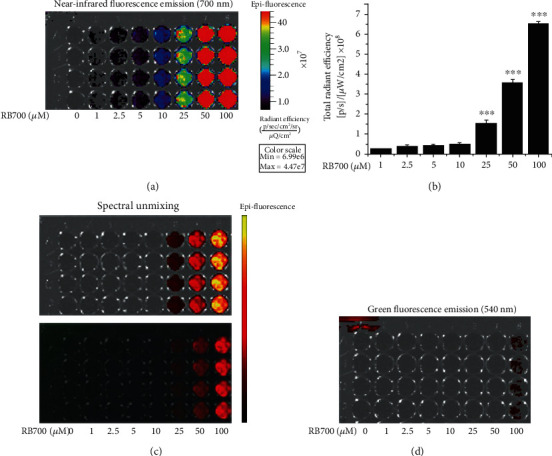
Determination of the optimal conditions for detection of the RB700 signal using the IVIS™ imaging system. RB700 (1-100 *μ*M) was oxidized in the presence of Fenton's reagent (10 *μ*M FeSO_4_ + 100 *μ*M H_2_O_2_) for 1 minute. (a) A representative image of epifluorescent emission is shown in panel. (b) Quantitative detection of the signal generated by the increasing concentrations of oxidized RB700. *n* = 4, ^∗∗∗^*P* < 0.001. Statistical *P* values were calculated relative to the level of fluorescence associated with the 1 *μ*M of oxidized RB700. (c) Spectral analysis by removing the background signal that highlights the specificity of the RB700 fluorescence emission in the near-infrared range. (d) Representative image demonstrating the absence of RB700 emission in the green spectral range is shown in panel.

To set up the optimal excitation/emission fluorescence filter systems for the detection of the oxidized RB700 specific signal, test tube chemical reaction-based experiments were performed using the IVIS™ Spectrum Caliper 200 imaging system. To increase the RB700-dependent near-infrared fluorescence emission, RB700 (1 to 100 *μ*M) was oxidized for 1 minute in the presence of Fenton's reagent (10 *μ*M FeSO_4_ + 100 *μ*M H_2_O_2_), a chemical reaction that generates HO^·^, a highly reactive and toxic O_2_-derived free radical that initiates and amplifies the process of lipid peroxidation in atherogenesis. Test results revealed a progressive RB700 concentration-dependent upregulation of the total radiant efficiency, a hallmark of increased RB700 oxidation. Significant signal intensifications were generated by RB700 at the concentration of 25 *μ*M (≈1.5-fold), 50 *μ*M (≈3.5-fold), and 100 *μ*M (≈6.5-fold) (Figures 4(a)–4(c)). No oxidized RB700-related signal was detected at 540 nm emission wavelength, characteristic for green fluorescence emission of NBD-DSPE used to label the liposomes' membranes (Figure 4(d)). Collectively, the experimental results presented above recommend the use of RB700 in the concentration range of 25 to 100 *μ*M for optimal detection of ROS.

### 3.6. VCAM-1-Targeted Lp Efficiently Localize at the Sites of Aortic Atherosclerotic Lesions

**Figure 5:**
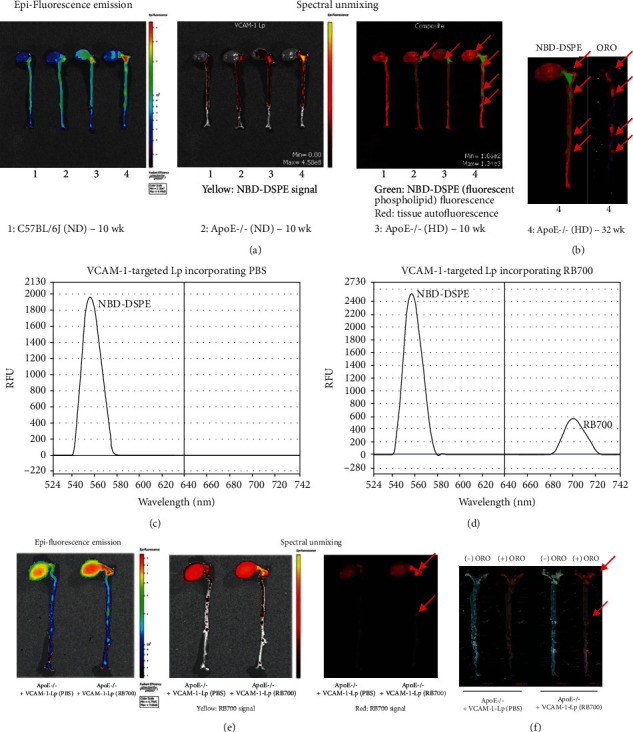
Analysis of VLp-RB700 targeted delivery of RB700 into the aortic atherosclerotic lesions in ApoE-/- mice. Fluorescently labeled VLp-RB700 were administrated to mice via retroorbital injection, and after 2 hours, the mice were sacrificed and the aortas analyzed using the IVIS™ imaging system. (a) Representative images of the epifluorescence signal induced by NBD-DSPE are shown, indicating the concentration of RB700 within the atherosclerotic lesions. Spectral unmixing analysis was done to delineate the specific NBD-DSPE signal (green) from the tissue autofluorescence (red). Arrows indicate the specific signal of NBD-DSPE. (b) Colocalization (indicated by arrows) of the labeled RB700-Lp with the atherosclerotic lesions assessed by mirroring of the NBD-DSPE signal with the oil red O (ORO) staining of the atherosclerotic aorta. (c, d) Representative spectral analysis of the VCAM-1-targeted Lp incorporating PBS solution or RB700. Note the emission spectra generated by Lp loaded with PBS (maximum emission peak in the green spectral range) and Lp loaded with RB700 (one maximum emission peak in the green spectral range related to NBD-DSPE and one maximum emission peak in the near-infrared spectral range related to RB700 are observed). (e) Comparative spectral analysis between aortas derived from atherosclerotic ApoE-/- mice injected with Lp (PBS) or Lp (RB700) shows that the localization of the oxidized RB700 near-infrared fluorescence signal along the aorta. (f) Representative images depicting the coincidence (arrows) between VLp-RB700 signal with the ORO staining of the aortic atherosclerotic lesions.

To test if VLp-RB700 is taken up efficiently and can be localized within the atherosclerotic lesions, the *ex vivo* analysis of the green signal coming from NBD-DSPE-labeled Lp was measured 2 hours after retroorbital administration of VLp-RB700 in mice. The results showed that the green fluorescence was predominantly concentrated in areas where atherosclerotic plaques develop, namely, the aortic arch (ApoE-/- (ND) and ApoE-/- (HD) mice, 10 weeks)) and throughout the aorta of ApoE-/- mice with advanced atherosclerosis (i.e., ApoE-/- (HD), 32 weeks) (Figures 5(a) and 5(b)). The specific emission spectra of NBD-DSPE-labeled Lp loaded with PBS or RB700 are depicted in Figures 5(c) and 5(d), respectively. Note that the two fluorescent compounds, NBD-DSPE and RB700, display different fluorescent emission spectra that do not overlap. No specific RB700 signal was detected in the near-infrared spectral range in Lp-encapsulated PBS-injected mice, as compared to mice that were injected with Lp-encapsulated RB700 (Figure 5(e)). A representative mirroring analysis of the RB700 signal with the ORO staining of lipids within aortic atherosclerotic lesions is shown in Figure 5(f). Collectively, the data demonstrate that sterically stabilized VCAM-1-targeted Lp is a reliable and straightforward strategy to specifically target the redox-sensitive probe RB700 to vascular territories with atherosclerotic lesions.

### 3.7. Colocalization of ROS Overproduction with Atherosclerotic Lesions in the Aorta of ApoE-/- Mice

**Figure 6:**
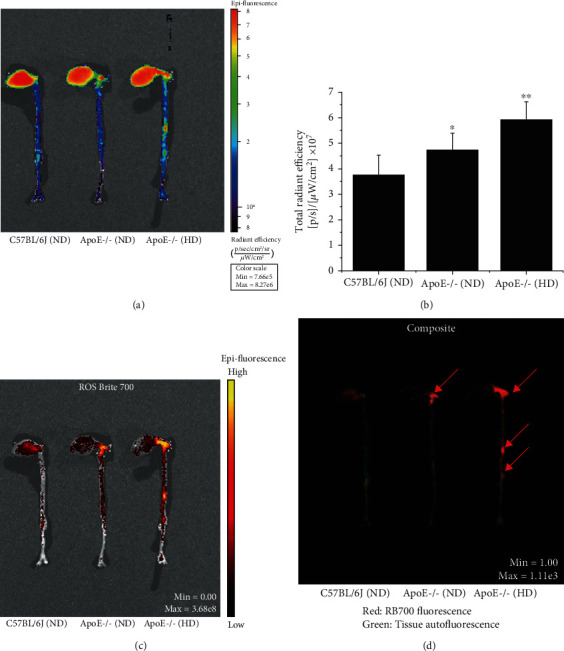
Colocalization of ROS production with the lesions in the atherosclerotic aorta of the ApoE-/- mice. VLp-RB700 were delivered via retroorbital injection to C57BL6/J (control) and ApoE-/- mice fed a normal diet (ND) or high-fat diet (HD) for 10 weeks. After 2 hours, the animals were sacrificed and the fluorescent signal of the oxidized RB700 was monitored *ex vivo* along the aortic segment using a high-resolution fluorescence imaging system. (a, b) Quantitative analysis of epifluorescent emission throughout the aorta of experimental mice. (c, d) Spectral unmixing analysis was performed to delineate the oxidized RB700-derived specific fluorescence signal from the tissue autofluorescence. The colocalization of ROS production with atherosclerotic lesions is depicted by arrows. *n* = 4-5, ^∗^*P* < 0.05, ^∗∗^*P* < 0.01. Statistical *P* values were calculated relative to the values obtained for C57BL6/J nonatherosclerotic control mice.

To localize ROS formation within atherosclerotic lesions, VLp-RB700 were administrated systemically to anesthetized mice via retroorbital injection. After 2 hours, the animals were sacrificed and the entire aorta along with the heart and various organs were harvested for the analysis of the fluorescent signal induced by the oxidation of RB700 in the near-infrared spectral range. A significant upregulation of total radiant efficiency was detected in the aorta of ApoE-/- (ND, 10 weeks, ≈1.25-fold) and ApoE-/- (HD, 10 weeks, ≈1.6-fold) mice featuring aortic atherosclerotic lesions as compared with C57BL6/J (ND) control animals. The fluorescence signal was mainly concentrated in areas where atherosclerotic plaques develop, namely, the aortic arch of ApoE-/- (ND) mice and throughout the aorta of ApoE-/- (HD) mice (Figures 6(a) and 6(b)). In addition, the spectral unmixing analysis revealed that the intensity of RB700 oxidation is maximal within the atherosclerotic plaques (Figures 6(c) and 6(d)).

**Figure 7:**
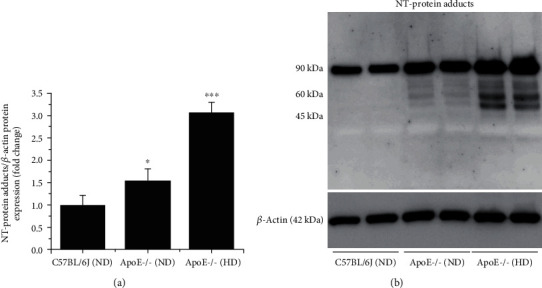
The levels of the oxidative stress marker, nitrotyrosine (NT), are significantly elevated and correlate well with the severity of the atherosclerotic lesions in the aorta of ApoE-/- mice. Total aortas harvested from C57BL6/J (control) and ApoE-/- mice fed a ND or HD for 10 weeks were subjected to Western blot using a specific anti-NT antibody. (a) Quantitative analysis of covalently modified protein levels in the presence of NT. (b) Representative immunoblot showing the accumulation of NT-modified proteins in the aorta of ApoE-/- mice. *n* = 4, ^∗^*P* < 0.05, ^∗∗∗^*P* < 0.001. *P* values were taken in relation to the values obtained in controls, C57BL6/J mice.

Based on the fact that RB700 is oxidized by O_2_^·-^ to produce bright near-infrared fluorescence emission, the formation of nitrotyrosine (NT), a stable and reliable marker of oxidative stress resulting from chemical reaction between O_2_^·-^ and NO, was measured in the aortic homogenates of mice by the Western blot assay. We detected a significant increase in oxidatively modified proteins by NT (i.e., NT-protein adducts) in the protein extracts derived from the aortas of ApoE-/- animals fed a normal diet (≈1.5-fold) or atherogenic diet (≈3-fold) as compared with the level obtained for controls, the C57BL6/J mice fed a normal diet for 10 weeks (Figures 7(a) and 7(b)).

**Figure 8:**
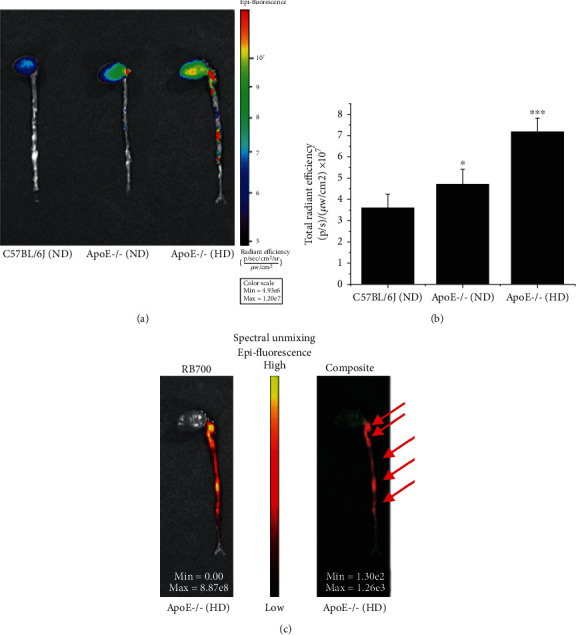
Colocalization of ROS-induced RB700 oxidation with the advanced plaque in the atherosclerotic aorta of ApoE-/- mice. C57BL/6J mice (controls, normal diet/ND, 10 weeks), ApoE-/- mice (ND, 10 weeks), and ApoE-/- that received high-cholesterol diet (HD) for 32 weeks were injected retroorbital with VLp-RB700, and after 2 hours, the animals were sacrificed, and the aortas collected and analyzed using the IVIS™ system. (a, b) Quantitative analysis of epifluorescent emission throughout the aorta of experimental mice. (c) The colocalization of the oxidized RB700 fluorescence signal after spectral unmixing at the level of atherosclerotic lesions (arrows) present along the aorta of ApoE-/- mice fed with the atherogenic diet for 32 weeks is observed. *n* = 4, ^∗^*P* < 0.05, ^∗∗∗^*P* < 0.001. The statistical value *P* was calculated in relation to the C57BL6/J (ND) level.

The localization of ROS within advanced atheroma was further investigated in ApoE-/- mice fed a HD for 32 weeks. *Ex vivo* analysis of the fluorescence signal, measured 2 hours after administration of VLp-RB700, demonstrated significant increases in RB700 oxidation along the atherosclerotic aorta of ApoE-/- (ND, ≈1.3-fold) and ApoE-/- (HD, ≈2-fold) mice as compared with the levels detected in the aorta of C57BL6/J (ND), control animals (Figures 8(a) and 8(b)). The spectral unmixing analysis revealed that the intensity of RB700 oxidation is maximal within the atherosclerotic plaques (Figure 8(c)).

### 3.8. Oxidation of RB700 Is Significantly Increased in the Organs of Atherosclerotic ApoE-/- Mice

**Figure 9:**
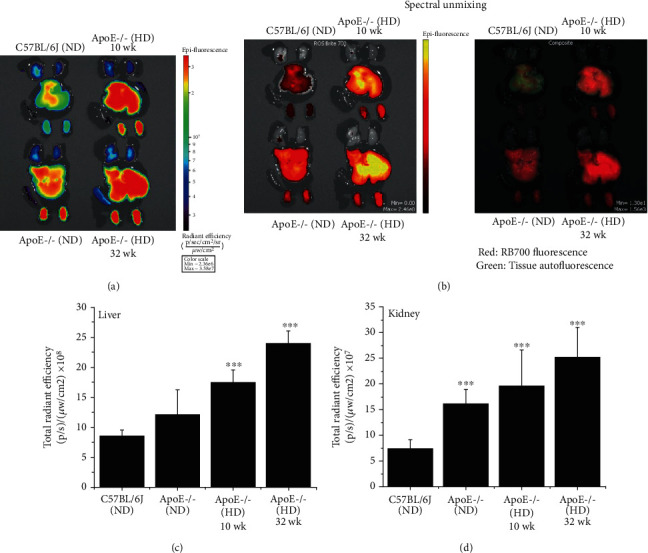
The fluorescence signal of oxidized RB700 is significantly increased in the liver and kidney of atherosclerotic ApoE-/- mice as compared with C57BL6/J and ApoE-/- mice maintained on ND. After 2 hours, the animals were sacrificed and the organs (lung, liver, spleen, and kidney) were analyzed using the IVIS™ system. (a, b) Analysis of the epifluorescence emission signal of the oxidized RB700. (c, d) Quantitative analysis of the epifluorescence emission signal of the oxidized RB700 in the liver and kidneys of mice following spectral unmixing analysis to eliminate the tissue autofluorescence. *n* = 3-6/animals group, ^∗∗∗^*P* < 0.001. The statistical *P* values were calculated in relation to the C57BL6/J (ND) level.

The analysis of the ROS formed *in vivo* followed by *ex vivo* detection of ROS-induced RB700 oxidation was further extended to various organs (e.g., lung, liver, spleen, and kidneys). As depicted in Figure 9, the specific near-infrared fluorescent signal of the oxidized RB700 was found significantly elevated in the liver and the kidneys of ApoE-/- mice maintained for 10 or 32 weeks on an atherogenic diet as compared to controls, C57BL/6J and ApoE-/- mice fed a normal diet. These data indicate that RB700 encapsulation into sterically stabilized VCAM-1-sensitive Lp could be an important strategy for *ex vivo* detection of the ROS formed *in vivo* in various organs and tissues in different experimental animal models of disease.

## 4. Discussion

In spite of the significant advancement in the understanding of the regulatory pathways and molecular sources of ROS formation and function in different biological systems, the specific detection and quantification of ROS remain a challenge [13, 14]. Since oxidative stress is instrumental in the pathoetiology of numerous human maladies, an increasing interest is shown towards methods and tools to specifically address the role of ROS in different *in vitro* and *in vivo* experimental models of disease, the results of which could be translated to human maladies. A wide range of colorimetric, fluorometric, chemiluminescent, electron spin resonance, magnetic resonance, electrochemical, ultrasound-based, and immunospecific assays have been developed in an attempt to distinctively detect, localize, and quantify various types of ROS in a wide range of experimental settings [15–24]. However, because of the unique chemical characteristics of ROS, all the current methods for the specific interrogation of its production have both strengths and limitations [17].

Due to the molecular attributes of ROS, the development of reliable detection methods implies the design of highly specific redox-sensitive probes that would compete with the cellular enzymatic/nonenzymatic systems and molecular components to produce a stable measurable product upon ROS oxidation [13–16]. Yet, some redox-sensitive probes have a limited capacity to trace ROS formation in both extracellular and intracellular compartments [15–17]. Moreover, recapitulating the dynamic and complex *in vivo* conditions in a test tube is even more challenging. Thus, even today, the detection of the ROS formed *in vivo* is a major goal in free radical biology research.

Over the past two decades, several ROS-sensitive fluorescent probes for monitoring ROS production in living cells have been developed. These include dihydrochlorofluorescein-diacetate, dihydrorhodamine, dihydroethidium, and HyPer family probes [25–35]. The advantages of using fluorescent probes include high sensitivity and method simplicity that allow direct quantification and localization of the sites of ROS formation within cell compartments. Some of these probes, including dihydroethidium, have been adapted to assess the potential of biological samples, typically frozen cryosections, to produce ROS, *ex vivo* [3]. Yet, the detection of the ROS generated *in vivo* employing fluorescent-based probes remained challenging.

Thus, in this study, we developed a straightforward method employing targeted Lp-mediated delivery of RB700, a cell-impermeant fluorogenic probe, to directly localize and quantify ROS production in atherosclerotic lesions and organs in experimental hypercholesterolemia. The *ex vivo* analysis of the ROS-induced RB700 oxidation signal was employed using the high-resolution fluorescence-based IVIS™ imaging system. Notably, RB700 probe produces bright near-infrared fluorescence upon O_2_^·-^ and HO^·^ oxidation [36, 37], two major ROS with an important role in the pathogenesis of atherosclerosis. Another major advantage of this method is that it enables the detection of the ROS formed *in vivo* and it does not reflect only the potential of the samples to generate ROS, as in the case of a wide number of assays employing tissue homogenates or cryosections [15–17].

RB700 has been used *per se* as a ROS-selective probe in several *in vitro* and *in vivo* studies to monitor the overall formation of O_2_^·-^ and HO^·^. It was shown that RB700 is a reliable probe in assessing ROS formation in a tissue homogenate detection system [38]. Moreover, intravenous administration of RB700 and full-body fluorescence imaging was used to monitor ROS overproduction in a mouse model of colorectal cancer [39]. Yet, RB700 is a cell-impermeant compound, and therefore, in the absence of an intracellular delivery system of RB700, critical ROS-related processes occurring within cells or cell compartments as well as the contribution of numerous ROS-generating systems remain underestimated.

Different formulations of Lp have been extensively used for targeted drug delivery in a large number of biological systems [40–42]. Noteworthily, a specific formulation of VCAM-1-targeted Lp used to block site-specific chemokine-related inflammatory processes in vascular diseases and cancer metastasis has been successfully developed by our group [8]. Thus, in an attempt to overcome the technical limitation generated by the cell-impermeant attribute of RB700, we customized the previously designed and optimized specific formulation of Lp to encapsulate RB700, this ROS-specific fluorogenic probe, and thus to facilitate the intracellular delivery. Initially, we determined, on cultured EC and Mac, an efficient cellular uptake of the Lp incorporating RB700 with no cytotoxic effects. Then, to enable the targeted delivery of RB700 at the sites of atheroma, peptides that specifically recognize VCAM-1, a molecule expressed by the dysfunctional endothelium in atherosclerosis, were conjugated on the surface of the Lp containing PEG-derivatized phospholipids. Notably, targeting peptides that specifically recognize VCAM-1 rather than antibodies were used in order to minimize the potential host immune responses and adverse effects. The results demonstrated that 2 hours after systemic administration to atherosclerotic ApoE-/- mice, VCAM-1-targeted Lp (VLp-RB700) were significantly concentrated at the sites of atherosclerotic lesions and were significantly correlated with the severity of the disease. Moreover, in the ApoE-/- mice featuring intermediate and advanced atherosclerotic lesions throughout the aorta, a robust upregulation of the oxidized RB700 fluorescence signal was determined in the liver, the major organ producing ROS in the body, and in the kidneys.

Collectively, our data indicate that the encapsulation of RB700 within endothelium-targeted sterically stabilized Lp could become a reliable method to directly quantify and visualize the sites of ROS formation in the vasculature and organs in small animal models of disease. The method could be applied not only for experimental atherosclerosis but also for cancer, diabetes, obesity, and neurodegenerative diseases, since all these maladies are marked by enhanced ROS formation. Moreover, this novel set-up for the molecular detection of ROS based on activated endothelium-targeted RB700-Lp is of particular importance for understating the intensity and the mechanism(s) of risk factor-induced oxidative stress associated with different pathologies, as well as for the preclinical development and test of new drugs and therapeutic strategies.

## Data Availability

The data used to support the findings of this study are available from the corresponding authors upon request.

## Abbreviations

ROS: Reactive oxygen species
RB700: ROS Brite™ 700
Lp: Liposome
VLp: VCAM-1-targeted, sterically stabilized liposome
VLp-RB700: VLp incorporating RB700
NBD-DSPE: 1,2-Distearoyl-sn-glycero-3-phosphoethanolamine-N-(7-nitro-2-1,3-benzoxadiazol-4-yl)
DPPC: 1,2-Dipalmitoyl-sn-glycero-3-phosphocholine
DOPA: 1,2-Dioleoyl-snglycero-3-phosphatidic acid
NBD-PE: 1,2-Dipalmitoyl-sn-glycero-3-phosphoethanolamine-N-(7-nitro-2-1,3-benzoxadiazol-4-yl) (ammonium salt)
PEG: *Polyethylene glycol*
PEG-DSPE: 1,2-Distearoyl-sn-glycero-3-phosphoethanolamine-N-[methoxy (polyethylene glycol)-2000] (ammonium salt)
Mal-PEG-DSPE: 1,2-Distearoyl-sn-glycero-3-phosphoethanolamine-N-[maleimide (polyethylene glycol)-2000]
UHPLC: Ultra-high-performance liquid chromatography
PDI: Particle size and polydispersity index
DLS: Dynamic light scattering.
